# Supplementary material for: Relation of Minimally Processed Foods and Ultra-Processed Foods with the Mediterranean Diet Score, Time-Related Meal Patterns and Waist Circumference: Results from a Cross-Sectional Study in University Students
Source: Int J Environ Res Public Health. 2023 Feb 4;20(4):2806. doi: 10.3390/ijerph20042806 (PMC9957033; doi:10.3390/ijerph20042806)

Supplementary Figure S1: Scatter dot plots between UPF and WC, BMI in the total sample, men and women

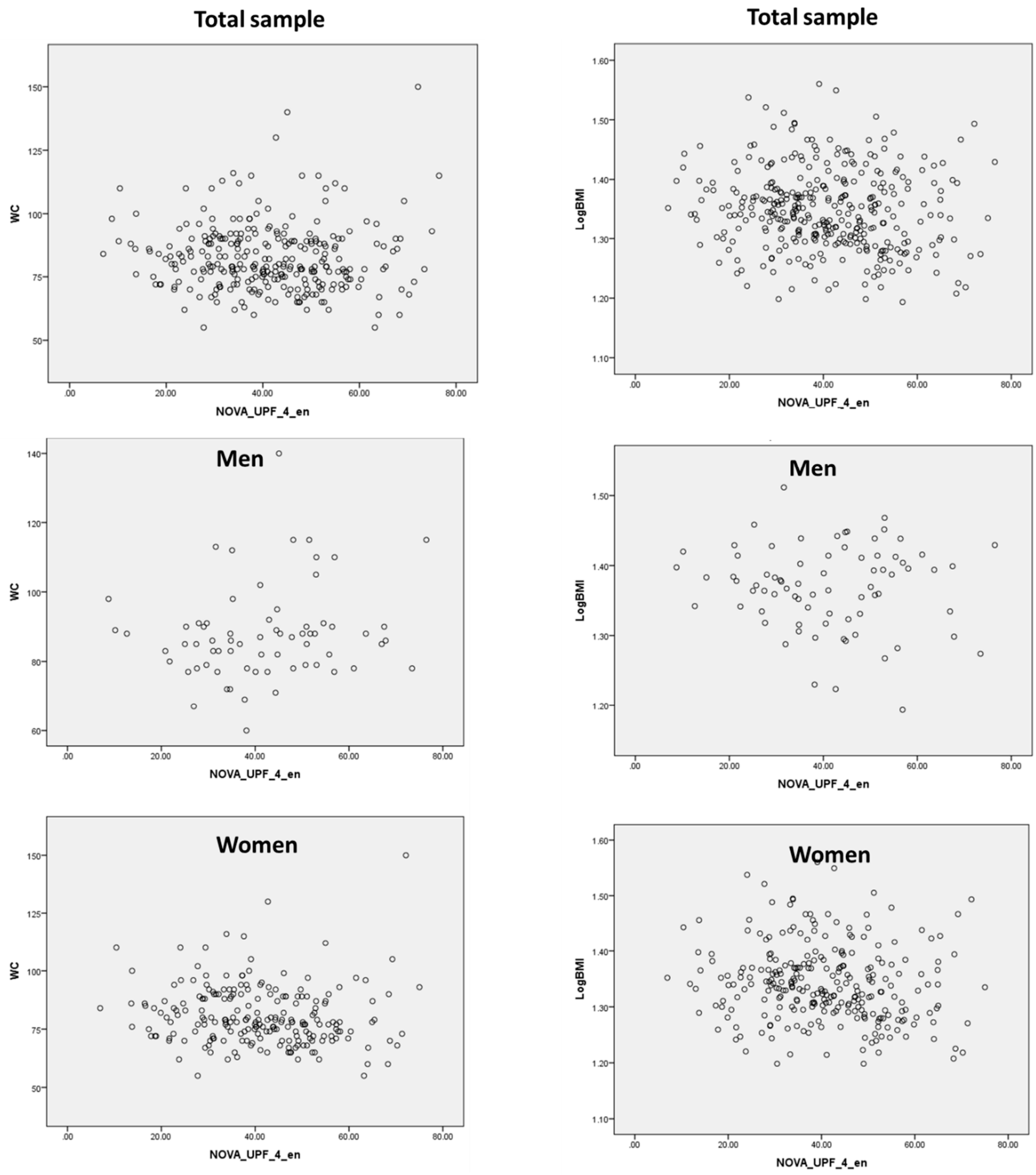

Supplementary Figure S2: Scatter dot plots between UPF and MedDietScore in the total sample, men and women.

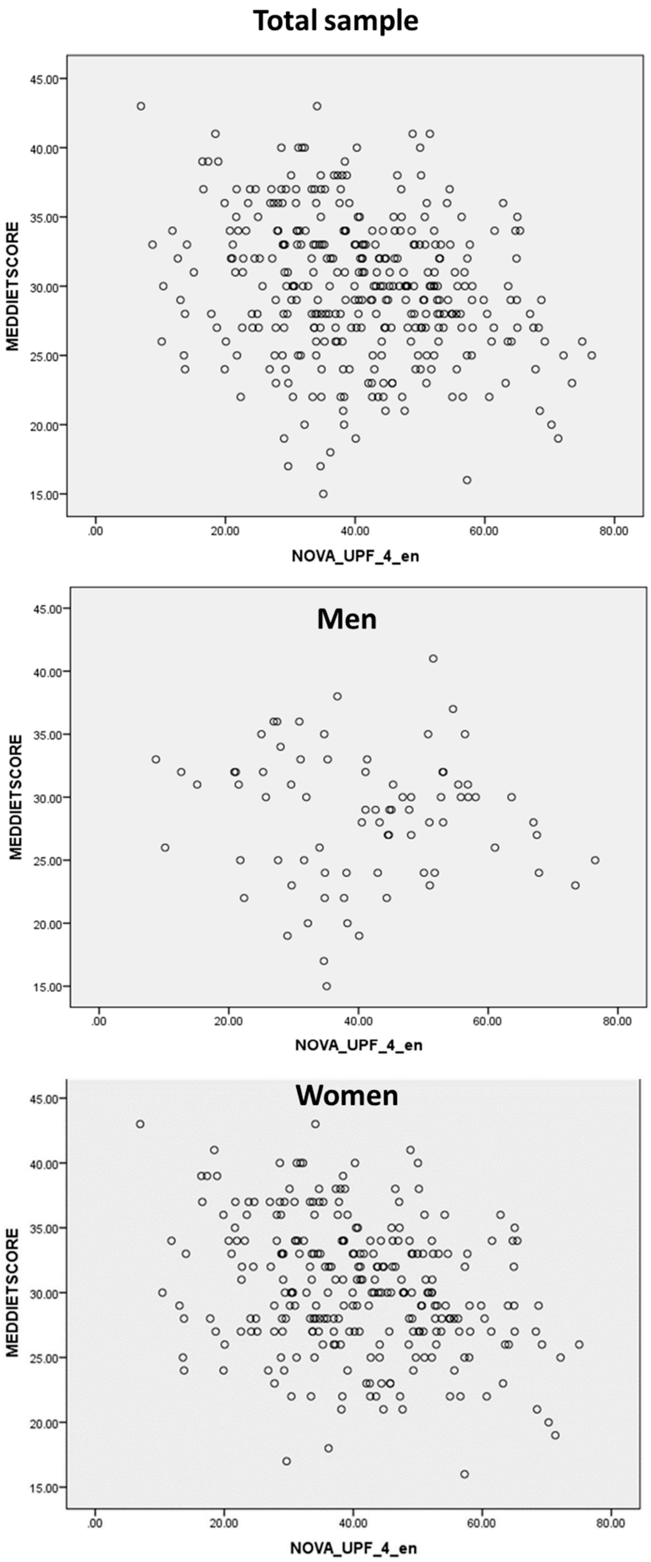

Supplementary Figure S3: Scatter dot plots between UPF and early, late eating patterns in the total sample, men and women.

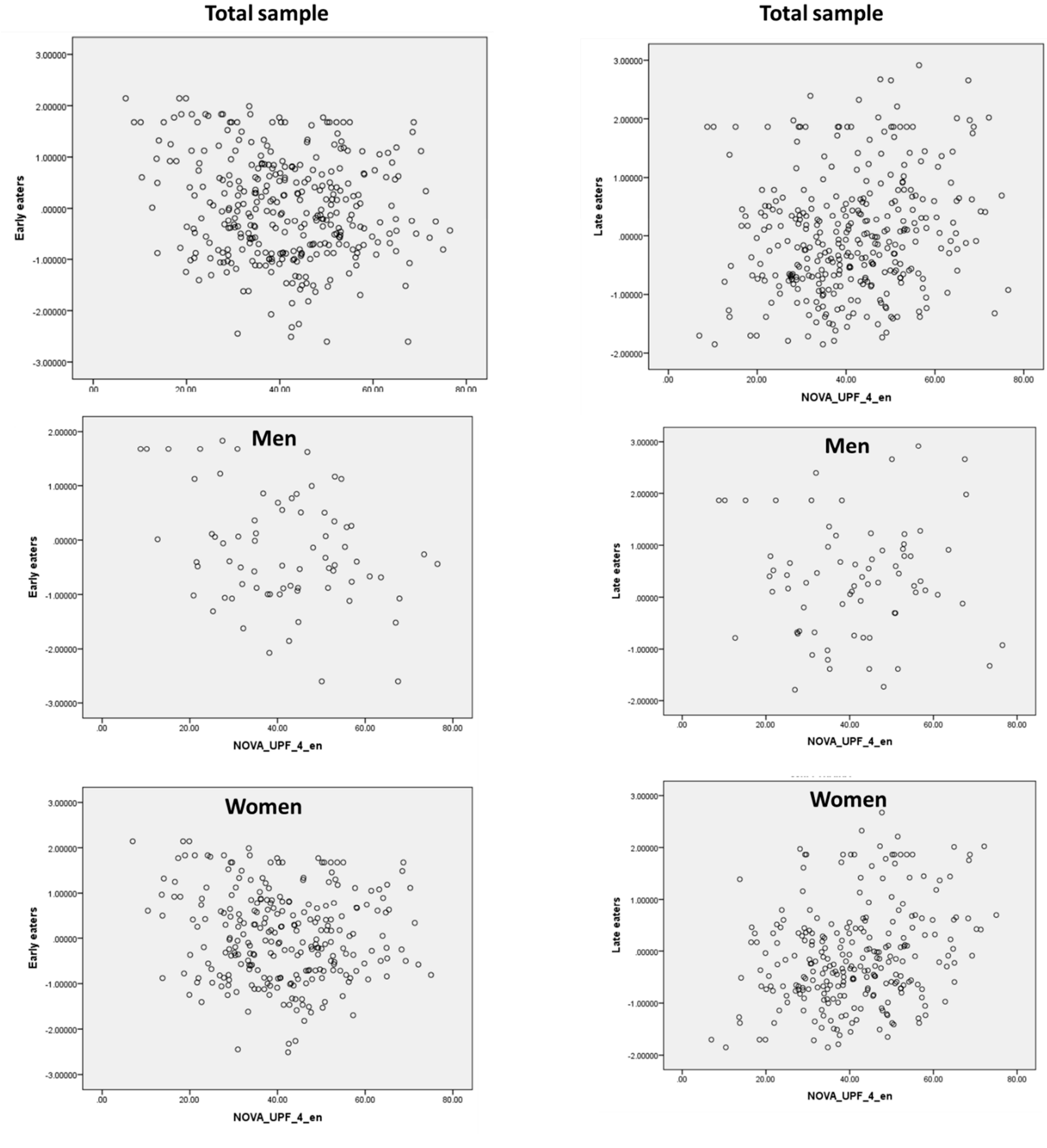

Supplement: Supplementary file 1 [file ijerph-20-02806-s001.zip › Supplementary Figures.pdf]
