# Supplementary material for: Relation of Minimally Processed Foods and Ultra-Processed Foods with the Mediterranean Diet Score, Time-Related Meal Patterns and Waist Circumference: Results from a Cross-Sectional Study in University Students
Source: Int J Environ Res Public Health. 2023 Feb 4;20(4):2806. doi: 10.3390/ijerph20042806 (PMC9957033; doi:10.3390/ijerph20042806)
Supplement: Supplementary file 1 [file ijerph-20-02806-s001.zip › Supplementary Table S1.pdf]

Supplementary Table S1: Additional linear regression analysis for l/WC in the total sample, men and women with MedDietScore and eating patterns as covariates.

Dependent variable: 1/WC	Total sample<br>adj R^2= 18.3%						Men						Women					
	B	SE	p	Tole-rance	VIF	adj R^2 (%)	B	SE	p	Tole-rance	VIF	adj R^2 (%)	B	SE	p	Tolerance	VIF	adj R^2 (%)
Model 1: Basic Model	3.6×10^{-6}	5×10^{-6}	0.645	0.935	1.070	18.3	-3×10^{-5}	10^{-5}	0.050	0.807	1.239	22.3	1.5×10^{-5}	10^{-5}	0.188	0.949	1.054	11.1
Model 2: Basic Model +MedDietScore	-1.05×10^{-6}	10^{-5}	0.911	0.857	1.167	12.7	-3.20×10^{-5}	1.7×10^{-5}	0.059	0.882	1.134	19.2	-3.36×10^{-5}	3.1×10^{-5}	0.284	0.797	1.255	8.1
Model 3: Basic Model + early/ late eating patterns	2.16×10^{-6}	10^{-5}	0.824	0.829	1.206	12.8	-2.64×10^{-5}	1.9×10^{-5}	0.175	0.606	1.651	14.2	1.22×10^{-5}	1.2×10^{-5}	0.295	0.813	1.230	7.0
Model 4: Basic Model+ MedDietScore+ early/ late eating patterns	-1.51×10^{-6}	10^{-5}	0.881	0.778	1.286	13.3	1.94×10^{-6}	4.5×10^{-5}	0.184	0.591	1.692	11.9	7.17×10^{-6}	1.2×10^{-5}	0.564	0.719	1.390	7.3

B unstandardised coefficients, SE, p-values, tolerance and VIF are displayed for total UPF (% of total energy). The adjR^2 is reported for the whole model.
Basic model was adjusted for age, sex, department, living area and underreporting and is the same reported in Table 3.
Early/late eating patterns were entered alltogether (early/medium/late eating pattern).
